# Serum Outperforms Plasma for Glypican-3 Quantification in Hepatocellular Carcinoma—A Prospective Comparative Study

**DOI:** 10.3390/jcm15020448

**Published:** 2026-01-07

**Authors:** Ming-Tze Yang, Jiunn-Min Wang, Chen-Shiou Wu, Shou-Wu Lee, Hsin-Ju Tsai, Chia-Chang Chen, Ying-Cheng Lin, Hui-Fen Liu, Teng-Yu Lee

**Affiliations:** 1 Department of International Medical Service Center, Taichung Veterans General Hospital, No. 1650, Sec. 4, Taiwan Boulevard, Taichung 40705, Taiwan; ming75@vghtc.gov.tw; 2 Department of Laboratory Medicine, Taichung Tzu Chi Hospital, Buddhist Tzu Chi Medical Foundation, Taichung 42743, Taiwan; tc23112803@tzuchi.com.tw (J.-M.W.); tc151022@tzuchi.com.tw (H.-F.L.); 3 Department of Medical Research, Taichung Veterans General Hospital, No. 1650, Sec. 4, Taiwan Boulevard, Taichung 40705, Taiwan; chanshu65@gmail.com; 4 Division of Gastroenterology and Hepatology, Department of Internal Medicine, Taichung Veterans General Hospital, No. 1650, Sec. 4, Taiwan Boulevard, Taichung 40705, Taiwan; ericest@vghtc.gov.tw (S.-W.L.); a9194024@hotmail.com (H.-J.T.); jcchen@vghtc.gov.tw (C.-C.C.); ethankevin516@gmail.com (Y.-C.L.); 5 School of Medicine, Chung Shan Medical University, Taichung 40201, Taiwan; 6 Department of Post-Baccalaureate Medicine, National Chung Hsing University, Taichung 40227, Taiwan

**Keywords:** biomarker, liver cancer, sensitivity, specificity, storage condition

## Abstract

**Background:** Glypican-3 (GPC3) is frequently overexpressed in hepatocellular carcinoma (HCC) and serves as a circulating biomarker. Limited evidence exists regarding whether plasma or serum constitutes the optimal matrix for GPC3 measurement. This study aimed to investigate this gap. **Methods:** Between December 2024 and September 2025, 100 participants were prospectively enrolled, including 33 healthy controls, 29 individuals with chronic liver disease, and 38 patients with HCC. Paired serum and plasma samples were analyzed under fresh conditions and after storage for seven days at 4 °C and −70 °C. GPC3 concentrations were compared across groups. Subsequently, correlation and area under the receiver operating characteristic curve (AUROC) analyses were conducted. **Results:** In fresh samples of the controls, median plasma GPC3 levels were significantly higher than those in serum (82.36 pg/mL, IQR: 67.56–92.42 vs. 30.89 pg/mL, IQR: 20.36–41.12; *p* < 0.001). After seven days of storage, plasma GPC3 concentrations declined markedly at both 4 °C (41.73 pg/mL, IQR: 32.49–55.37; *p* < 0.001) and −70 °C (45.53 pg/mL, IQR: 25.30–55.65; *p* < 0.001), with no significant difference between the two storage conditions (*p* = 0.610). In contrast, serum GPC3 levels remained relatively stable across fresh, 4 °C (31.10 pg/mL, IQR: 16.84–38.60), and −70 °C (25.31 pg/mL, IQR: 14.36–40.74) conditions (*p* = 0.645). Both matrices under −70 °C storage effectively discriminated HCC from non-HCC cases, although serum demonstrated a significantly better diagnostic performance (AUROC: 0.836, 95% CI: 0.749–0.902 vs. 0.772, 95% CI: 0.677–0.850; *p* = 0.013). **Conclusions:** Although plasma offers operational convenience and higher baseline GPC3 levels, serum provides both greater stability and superior diagnostic accuracy under frozen conditions, thus supporting its use as the preferred specimen matrix in clinical and research applications.

## 1. Introduction

Hepatocellular carcinoma (HCC) ranks amongst the leading causes of cancer-related mortality worldwide [1]. Despite advances in both surgical and non-surgical therapies, its marked biological heterogeneity means that a substantial proportion of patients are still diagnosed at the intermediate to advanced stages [2], rendering management particularly challenging. Early detection remains pivotal towards improving prognosis [3]. Current diagnostic and surveillance strategies rely primarily on imaging, including ultrasonography, computed tomography, and magnetic resonance imaging, and are often complemented by the measurement of serum alpha-fetoprotein (AFP) [3,4]. To address these challenges, the discovery and application of biomarkers have become central, enabling earlier detection, more accurate diagnosis, and individualized treatment planning. Several circulating biomarkers have shown promise, most notably AFP, protein induced by vitamin K absence or antagonist-II (PIVKA-II), and glypican-3 (GPC3), with accumulating evidence supporting their utility in both enhancing clinical management and improving outcomes [5,6].

GPC3 is a member of the heparan sulfate proteoglycan family and is predominantly expressed in the placenta and fetal liver, with minimal expression in normal adult tissues [7,8]; however, it is frequently and tumor-specifically overexpressed in HCC [9]. From a mechanistic perspective, GPC3 contributes to hepatocarcinogenesis through multiple pathways, including c-Myc, Wnt, IGF, YAP, and Hedgehog, and is involved in key tumorigenic processes such as tumor proliferation, metastasis, apoptosis resistance, epithelial–mesenchymal transition, and immune evasion, thereby reinforcing its biological rationale as both a diagnostic and therapeutic target [7,10]. GPC3 can be cleaved by furin at Arg358/Ser359, releasing soluble GPC3 into the circulation [7]; it has also been detected in small extracellular vesicles, supporting its feasibility as a blood-based biomarker [11]. Owing to its tumor-restricted expression profile and biological relevance, GPC3 has emerged as a promising target for both early detection and precision therapy. Prior studies indicate that serum GPC3 alone is generally inferior to AFP in differentiating HCC from liver cirrhosis, whereas its diagnostic performance is substantially improved when combined with AFP [12]. Moreover, serum GPC3 has been reported as an independent prognostic factor in patients with HCC, irrespective of tumor stage and response to therapy [13]. Furthermore, multiple GPC3-directed therapeutic strategies are currently under investigation, including bispecific and monoclonal antibodies, antibody-drug conjugates, peptide vaccines, and chimeric antigen receptor T-cell (CAR-T) approaches, as well as applications in molecular imaging [9,14,15,16].

Prior studies have shown that freezing, thawing, and storage duration can systematically affect measurements of blood protein biomarkers. Repeated freeze–thaw cycles can markedly interfere with protein assays and lead to declines in the concentrations of specific proteins [17]. Moreover, evaluating “freezing” and “thawing” as separate steps indicates that each is sufficient to perturb the composition of plasma metabolites and proteins [18]. In addition, many routinely measured serum analytes deviate from baseline after prolonged storage or multiple freeze–thaw cycles [19]. Taken together, these findings underscore the critical impact of pre-analytical conditions on liquid biopsy biomarker measurements. Plasma offers the procedural advantage of eliminating clotting time and thereby accelerating pre-analytical processing, but research on both the performance and stability of plasma-based GPC3 measurement remains limited. Moreover, direct comparative evaluations involving serum under different storage conditions are lacking. Based on these considerations, the present study systematically compared GPC3 concentrations, storage stability, and diagnostic performance between serum and plasma, with particular attention to fresh, 4 °C, and −70 °C storage conditions. This study aimed to clarify the feasibility of using plasma as an analytical matrix and to assess its relative advantages and limitations when compared with serum in the diagnostic evaluation of HCC.

## 2. Materials and Methods

### 2.1. Ethical Approval and Participants

This study was conducted in accordance with the Declaration of Helsinki and approved by the Institutional Review Board (IRB) of Taichung Veterans General Hospital (IRB No. CF21236B). Written informed consent was obtained from all participants prior to enrollment. Study participants included three groups: (1) healthy controls (HC) recruited during routine health examinations, (2) patients diagnosed with chronic liver disease (CLD) who were recruited from the outpatient clinic, and (3) patients with hepatocellular carcinoma (HCC) diagnosed according to established clinical and pathological criteria. Demographic and clinical data, including standard laboratory tests, were collected at the time of enrollment.

### 2.2. Sample Collection and Processing

Peripheral blood was obtained from all participants through standard venipuncture, and paired serum and plasma samples were prepared for each participant. Serum samples were collected in gel-separator tubes (Becton, Dickinson and Company, Columbus, NE, USA) and centrifuged at 3750 rpm for 10 min, after which the supernatant was collected for analysis using a biochemical immunoassay analyzer. Plasma samples were collected in lithium heparin anticoagulant tubes (Greiner Bio-One GmbH, Remsmünster, Austria) and immediately centrifuged at 3750 rpm for 10 min to remove cellular components, with all procedures performed in accordance with the manufacturers’ instructions. For stability assessment, the supernatant obtained after centrifugation was aliquoted into cryogenic tubes and stored at either 4 °C or −70 °C. Aliquots were analyzed either immediately or after controlled storage at 4 °C or −70 °C for up to 7 days prior to analysis. For each participant, paired serum and plasma were prepared. Serum was collected in yellow-top tubes and processed according to the manufacturer’s instructions. Plasma was collected in anticoagulant tubes and promptly centrifuged to remove cellular components. For stability assessments, aliquots were analyzed fresh or stored under controlled conditions at 4 °C or −70 °C for up to seven days prior to analysis.

### 2.3. Measurement of GPC3

Quantitative measurements of GPC3 levels in serum and plasma were performed using the CanAg Glypican-3 enzyme immunoassay kit (Fujirebio Diagnostics AB, Gothenburg, Sweden) following the manufacturer’s instructions. A solid-phase, two-step sandwich immunoassay was used, employing two mouse monoclonal antibodies against distinct epitopes of the GPC3 core protein. After sequential incubation involving a biotinylated antibody and a horseradish peroxidase-conjugated antibody, colorimetric detection was performed using 3,3′,5,5′-Tetramethylbenzidine substrate, with absorbance read at 450 nm. Calibration curves were generated from provided standards, and concentrations were calculated accordingly.

### 2.4. Statistical Analysis

Analyses were performed using MedCalc (version 20.015) and GraphPad Prism 8. Continuous variables are reported as the median with interquartile range (IQR). Between-group differences were tested with Kruskal–Wallis or one-way ANOVA (continuous) and chi-square (categorical). Diagnostic performance was evaluated through the area under the receiver operating characteristic curve (AUROC). Two-sided *p* < 0.05 was considered significant.

## 3. Results

### 3.1. Baseline Characteristics of Study Participants

A total of 100 individuals were enrolled, including 33 healthy controls, 29 patients with CLD, and 38 patients with HCC. As indicated in Table 1, significant differences were observed amongst the three groups in age and gender distribution. Body weight and BMI were significantly higher in the CLD group when compared with the others. Laboratory parameters showed group differences in AST, ALT, albumin, WBC, hemoglobin and platelet counts. Regarding lifestyle factors, alcohol consumption also differed significantly between the groups. In contrast, height, bilirubin, creatinine, and smoking status showed no significant differences. The patient characteristics were consistent with real-world clinical practice.

### 3.2. Serum vs. Plasma Stability

**Figure 1:**
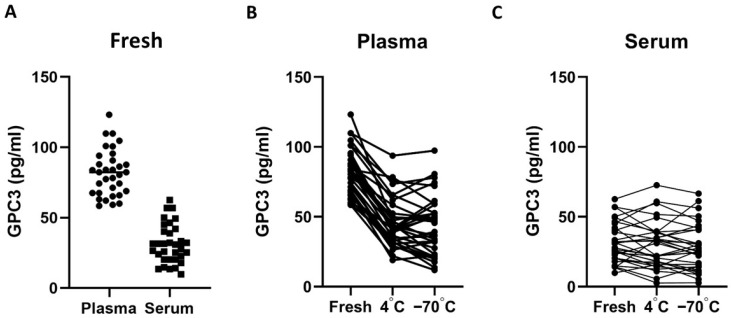
Comparison of GPC3 concentrations in plasma and serum samples under different storage conditions. (**A**) GPC3 concentrations in plasma and serum under fresh conditions. (**B**) Plasma GPC3 concentrations under fresh, 4 °C, and −70 °C storage conditions. (**C**) Serum GPC3 concentrations under fresh, 4 °C, and −70 °C storage conditions. GPC3, Glypican-3.

Amongst the healthy controls, we compared GPC3 concentrations in plasma and serum under three storage conditions: fresh, 4 °C, and −70 °C. As shown in Figure 1A, plasma GPC3 levels were consistently higher than those in serum, with a median concentration of 82.36 pg/mL (IQR: 67.56–92.42) compared to 30.89 pg/mL (IQR: 20.36–41.12; *p* < 0.001), reflecting an approximate 2.6-fold difference. However, as illustrated in Figure 1B, plasma GPC3 concentrations declined significantly during storage at 4 °C and −70 °C. After seven days of storage, plasma levels decreased to 41.73 pg/mL (IQR: 32.49–55.37; *p* < 0.001) at 4 °C and 45.53 pg/mL (IQR: 25.30–55.65; *p* < 0.001) at −70 °C, with no statistically significant difference being seen between the two storage conditions (*p* = 0.610). In contrast, as shown in Figure 1C, serum GPC3 concentrations remained relatively stable across all conditions: 30.89 pg/mL (IQR: 20.36–41.12) in fresh samples, 31.10 pg/mL (IQR: 16.84–38.60) after storage at 4 °C, and 25.31 pg/mL (IQR: 14.36–40.74) after storage at −70 °C (*p* = 0.645).

**Figure 2:**
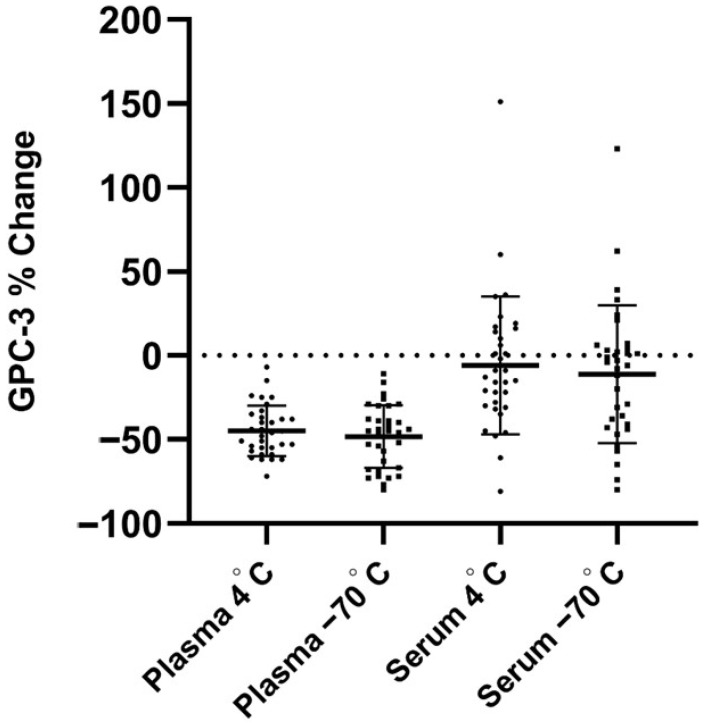
Percentage change in GPC3 concentrations in plasma and serum under different storage conditions when compared with fresh samples. Circles represent samples stored at 4 °C, and squares represent samples stored at −70 °C. GPC3, Glypican-3.

To further evaluate the stability of GPC3, we assessed the percentage change in concentration levels relative to freshly collected samples. As shown in Figure 2, plasma GPC3 levels exhibited a pronounced reduction following storage, with median decreases of −46% (IQR: −56 to −36) at 4 °C and −45% (IQR: −67.5 to −34) at −70 °C, indicating significant susceptibility to degradation. In contrast, serum GPC3 concentrations showed minimal variation, with median changes of −13% (IQR: −30.5 to 15) at 4 °C and −5% (IQR: −42 to 5) at −70 °C, suggesting substantially greater stability across storage conditions.

### 3.3. Correlation Analysis Between Plasma and Serum

**Figure 3:**
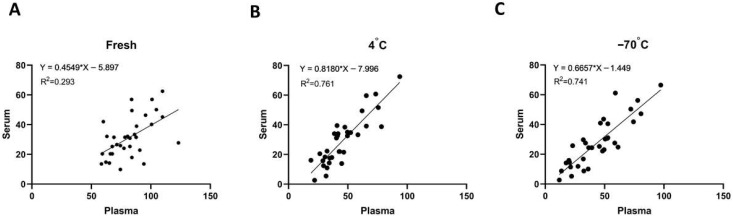
Correlation of plasma and serum GPC3 concentrations under different storage conditions. Scatter plots show the correlation between plasma and serum GPC3 levels in healthy subjects (*n* = 33). (**A**) Fresh samples. (**B**) Samples stored at 4 °C for seven days. (**C**) Samples stored at −70 °C for seven days. GPC3, glypican-3.

To further assess the relationship between plasma and serum GPC3 measurements, we conducted correlation analyses under different storage conditions (Figure 3A–C). In freshly collected samples, the correlation between plasma and serum GPC3 concentrations was moderate, exhibiting considerable variability (R^2^ = 0.293). However, following seven days of storage at either 4 °C or −70 °C, the correlation improved markedly, with higher R^2^ values observed at both 4 °C (R^2^ = 0.761) and −70 °C (R^2^ = 0.741). These findings indicate that storage stabilizes the relative relationship between plasma and serum GPC3 levels, leading to more consistent measurements across sample types. This enhanced concordance suggests that both plasma and serum may be suitable matrices for GPC3 quantification in both biomarker research and clinical applications, particularly under controlled storage conditions.

### 3.4. Comparison Amongst Clinical Groups

**Figure 4:**
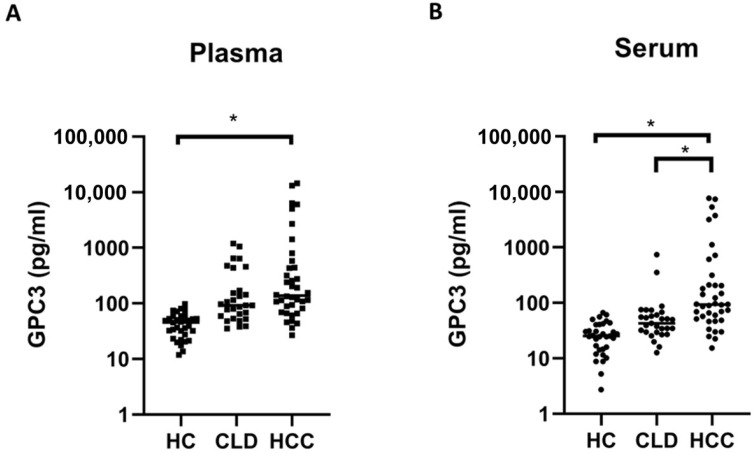
Comparison of GPC3 concentrations in plasma and serum across different clinical groups under −70 °C storage. (**A**) Plasma and (**B**) serum GPC3 levels were measured in HC, CLD, and HCC. Data are presented as scatter plots with mean values indicated. * *p* < 0.05. CLD, chronic liver disease; Glypican-3; HC, healthy control; HCC, hepatocellular carcinoma.

To further evaluate the clinical relevance of plasma versus serum GPC3 measurements, we compared concentrations among HC, individuals with CLD, and patients with HCC following storage at −70 °C. In plasma (Figure 4A), median GPC3 levels were significantly higher in the HCC group when compared to the HC group (137.6 pg/mL, IQR: 78.3–474.7 vs. 45.53 pg/mL, IQR: 25.30–55.65; *p* = 0.016). However, the difference between the HCC and CLD groups approached but did not reach statistical significance (137.6 pg/mL, IQR: 78.3–474.7 vs. 92.51 pg/mL, IQR: 56.23–303.7; *p* = 0.053). In contrast, serum GPC3 measurements (Figure 4B) showed significantly elevated levels in the HCC group (93.19 pg/mL, IQR: 50.70–236.8) when compared to both the HC group (25.31 pg/mL, IQR: 14.36–40.74; *p* = 0.013) and CLD group (42.76 pg/mL, IQR: 29.52–64.15; *p* = 0.027). While plasma measurements yielded higher absolute values and a broader dynamic range across groups, serum provided superior discriminatory performance for differentiating HCC from non-HCC (HC and CLD), whereas plasma showed only a borderline trend when distinguishing HCC from CLD.

### 3.5. Diagnostic Performance Analysis

**Figure 5:**
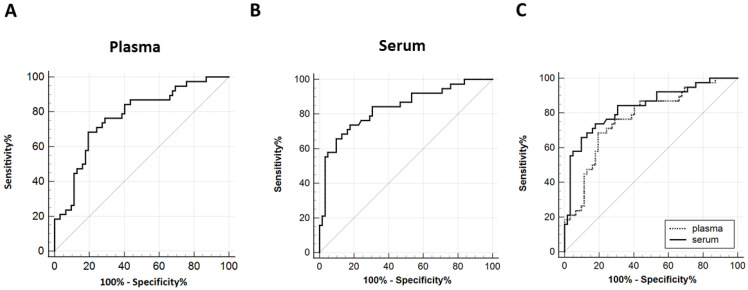
Diagnostic performance of plasma and serum GPC3 levels for discriminating HCC from non-HCC groups under −70 °C storage. AUROCs are shown for (**A**) plasma and (**B**) serum samples. (**C**) Direct comparison of plasma and serum GPC3 diagnostic performance under −70 °C storage. AUROC, area under the receiver operating characteristic curve; GPC3, Glypican-3; HCC, hepatocellular carcinoma.

To evaluate diagnostic utility under frozen storage, we performed ROC analyses. Plasma GPC3 achieved an AUROC of 0.772 (95% CI: 0.677–0.850) with an optimal cut-off of 106.76 pg/mL (Figure 5A), whereas serum GPC3 showed a higher AUROC of 0.836 (95% CI: 0.749–0.902) with a cut-off of 67.86 pg/mL (Figure 5B). As shown in Figure 5C, direct AUROC comparison confirmed serum’s superiority over plasma (*p* = 0.013). Collectively, although plasma yields higher absolute concentrations (Figure 4), serum provides more stable measurements and better discrimination between HCC and non-HCC, indicating that serum GPC3 under frozen storage is the more reliable biomarker for clinical application.

## 4. Discussion

To the best of our knowledge, this study is the first to systematically compare GPC3 concentrations, storage stability, and diagnostic performance between serum and plasma across three groups (healthy controls, CLD and HCC). We discovered that plasma generally exhibited higher absolute concentrations than serum yet was more sensitive to storage, with clear declines at both 4 °C and −70 °C, whereas serum GPC3 remained comparatively stable under the same conditions. Under frozen storage, serum also achieved a significantly higher AUROC than plasma, providing superior discrimination between HCC and non-HCC. Collectively, these findings indicate that serum GPC3 affords greater stability and reproducibility under frozen storage, reinforcing its role as the more reliable specimen type for clinical application and aligning with prior research that has primarily adopted serum as the standard matrix [20,21]. Although plasma may offer an advantage by eliminating clotting time and thus expediting pre-analytical processing, which in turn could enhance assay timeliness and accessibility in clinical settings, the findings of this study do not support its use as the preferred specimen matrix in clinical and research applications.

Previous investigations of GPC3 using plasma as the specimen matrix have been relatively limited, and reported results have been heterogeneous with respect to absolute concentrations and clinical performance [22,23]. In contrast, numerous positive findings from both laboratory and clinical studies support the use of serum as a specimen matrix for GPC3 measurement. Multiple meta-analyses and cohort studies have demonstrated that serum GPC3 is elevated in HCC and provides diagnostic accuracy comparable to AFP, with additional gains when used in combination panels [24]. In particular, combining serum GPC3 with AFP substantially improves sensitivity and overall diagnostic performance when compared with either marker alone [25]. Recent clinical series and reviews likewise support incorporating serum-based GPC3 into multi-marker algorithms (e.g., with AFP, PIVKA-II, AFP-L3) in order to improve early detection and prognostication [26,27]. Collectively, these findings indirectly support the validity of serum as an appropriate specimen matrix. In the present study, our paired analysis of serum and plasma provides important methodological insight. Although serum and plasma GPC3 levels were positively correlated, plasma GPC3 exhibited greater sensitivity to storage conditions, resulting in reduced stability and diminished post-storage performance. This matrix-dependent behavior suggests that cutoff values or diagnostic performance estimates derived from plasma cannot be directly extrapolated without careful harmonization of pre-analytical handling and storage conditions. Accordingly, comparisons with previously published plasma-based studies should prioritize relative diagnostic performance metrics, such as AUROC and incremental value within biomarker panels, rather than absolute concentration values. Importantly, these observations do not contradict prior plasma-based findings but instead provide essential context for their interpretation, while reinforcing serum as the more robust specimen matrix for applications requiring longitudinal stability and cross-study comparability.

The reasons for the greater sensitivity of plasma GPC3 to storage, with measurable declines observed at both 4 °C and −70 °C, warrant further discussion. Because short peptide fragments of soluble GPC3 are prone to proteolytic degradation, after seven days of storage, the measured concentration decreases by approximately 45%. By contrast, during serum formation, clotting removes a portion of clot-associated proteins and proteolytic enzymes, which may reduce protein breakdown and help maintain assay stability, thereby rendering plasma more vulnerable to storage-related loss [28]. Although plasma offers advantages in clinical workflows, such as eliminating clotting time and enabling faster processing, and our study also observed its generally higher absolute concentrations and wider dynamic range, which may help reduce false negatives, serum demonstrated greater stability and superior AUROC performance under frozen storage. This advantage is particularly important when tests need to be compared across different batches or over time, such as in multicenter studies, before and after comparisons, or biobank analyses. From a clinical interpretation perspective, integrating serum GPC3 with other serum-tested biomarkers such as AFP, PIVKA-II, or kininogen-1 could further enhance diagnostic sensitivity and specificity [20,25,29].

Beyond diagnostic applications, it is also critical to recognize the broader biological and therapeutic significance of GPC3. Several studies indicate that GPC3 is highly expressed in HCC tissues and is associated with an unfavorable prognosis [30,31]. For example, Xiao et al. reported that GPC3 overexpression is associated with more advanced tumor stage, vascular invasion, and poorer survival rates, affecting both overall and disease-free survival [31]. On the therapeutic front, GPC3-targeted antibody-drug conjugates [9], bispecific antibody strategies [32], and ongoing clinical trials of GPC3-directed CAR-T cells (e.g., NCT05003895, NCT06641453) collectively underscore the growing interest and potential of GPC3 as an immunotherapeutic target [33,34]. Taken together, GPC3 is a promising biomarker for early detection, prognosis prediction, and as a potential therapeutic target in HCC. A reliable blood matrix, such as serum, maintained under appropriate storage conditions may provide a valuable platform for GPC3 testing and contribute meaningfully to advancements in this field.

Several limitations within this study should be acknowledged. First, this study was conducted at a single center in Taiwan, which may limit the generalizability of the findings. However, while additional data from multicenter or international studies would be valuable for external validation, this prospective investigation clearly demonstrates key differences in GPC3 measurements between serum and plasma. Second, the overall sample size of the study was limited, and the number of patients within the HCC subgroup was relatively small, which may affect the precision and robustness of the HCC detection metrics. Although larger studies are still needed in order to confirm the comparative AUROC performance between serum and plasma, the observed stability of GPC3 concentrations in serum, particularly under the evaluated storage conditions, continues to support its use as the preferred specimen matrix. Third, the duration of blood sample storage may influence measured GPC3 concentrations; however, in this study, samples were evaluated only after a storage period of seven days. Nonetheless, serum and plasma GPC3 levels were compared under identical temperature and storage duration conditions, thereby ensuring that the primary objective of the study was appropriately addressed. Fourth, this study focused exclusively on GPC3, and other commonly used HCC biomarkers, such as AFP, were not evaluated in parallel. Future studies incorporating simultaneous comparisons of multiple biomarkers may yield more comprehensive and clinically informative insights.

In conclusion, this study demonstrated that although plasma GPC3 concentrations were markedly higher than serum concentrations in fresh samples and offered practical advantages by eliminating clotting time, plasma levels declined significantly after refrigeration or freezing, indicating greater sensitivity to storage conditions. In contrast, serum GPC3 remained relatively stable across storage conditions and exhibited superior diagnostic performance under −70 °C storage. Taken together, while plasma provides convenience in clinical workflows and retains reasonable diagnostic accuracy under frozen conditions, serum GPC3 offers greater stability and reliability, making it the preferred specimen type for both clinical testing and research applications.

## Figures and Tables

**Table 1:** Baseline demographics characteristics of study participants.

	Health Control (*n* = 33)	CLD (*n* = 29)	HCC (*n* = 38)	*p*
Age (years)	63.0 (50.0, 73.0)	55.0 (50.5, 64.5)	65.5 (58.8, 73.5)	<0.01
Male gender (*n*)	21	18	29	<0.01
Height (cm)	163.4 (156.7, 167.8)	165.0 (156.0, 170.5)	164.1 (158.6, 171.3)	0.53
Body weight (kg)	58.3 (49.5, 64.8)	70.8 (65.7, 78.1)	60.9 (53.8, 73.5)	<0.01
BMI (kg/m^2^)	20.9 (19.7, 24.5)	26.9 (23.4, 28.6)	23.1 (20.8, 25.4)	<0.01
Smoking (*n*)	0	3	2	0.18
Alcohol drinking (*n*)	0	10	15	<0.01
HBV infection (*n*)	0	10	18	<0.01
HCV infection (*n*)	0	2	5	<0.01
AST (U/L)	20.0 (17.5, 27.5)	44.0 (32.5, 66.0)	64.5 (35.5, 105.0)	0.15
ALT (U/L)	15.0 (11.0, 20.0)	44.0 (33.0, 87.0)	29.0 (17.0, 60.0)	<0.01
Bilirubin (mg/dL)	0.6 (0.5, 0.9)	0.8 (0.6, 1.3)	0.7 (0.6, 1.0)	0.89
Albumin (g/dL)	4.3 (4.1, 4.4)	4.6 (4.3, 4.8)	3.9 (3.3, 4.3)	0.11
Creatinine (mg/dL)	0.7 (0.6, 1.0)	0.8 (0.6, 0.9)	0.8 (0.6, 1.1)	0.05
White blood cell (mm^3^)	5470 (4765, 6428)	6180 (4760, 6815)	6515 (4918, 8608)	0.01
Hemoglobin (g/dL)	13.4 (12.6, 14.8)	14.1 (12.9, 15.7)	13.2 (10.4, 14.6)	<0.01
Platelet (mm^3^)	233.0 (181.0, 292.5)	234.0 (186.8, 267.8)	172.0 (116.3, 234.8)	<0.01

ALT, alanine aminotransferase; AST, aspartate aminotransferase; BMI, body mass index; CLD, chronic liver disease; HCC, hepatocellular carcinoma.

## Data Availability

Data are available upon request from the authors.
